# Elongated uvula and diagnostic utility of spirometry in upper airway obstruction

**DOI:** 10.4103/0970-2113.59266

**Published:** 2010

**Authors:** Rajiv Paliwal, Satish Patel, Purvesh Patel, Hiren Soni

**Affiliations:** *Department of Chest Medicine, P. S. Medical College, Karamsad, Anand, Gujarat - 388 315, India*; *Department of ENT, P. S. Medical College, Karamsad, Anand, Gujarat - 388 315, India*

**Keywords:** Elongated uvula, flow volume loops, upper airway obstruction

## Abstract

Elongated uvula is relatively an uncommon condition. Upper airway obstruction is often a missed complication of such a rare condition. Clinical presentations of upper airway obstruction often mimic asthma. Hence it is very easily mis-diagnosed as asthma. Spirometry offers a very simple test to diagnose upper airway obstruction very early and easily. Once diagnosed, the management of elongated uvula, almost exclusively, is surgical excision leading to total cure. Here is a case report of such a rare condition.

## INTRODUCTION

Disorders affecting upper airways are considerably less common than lower respiratory tract diseases but definitely not less important for several reasons. Patients presenting with obstructive symptoms are often misdiagnosed and unsuccessfully treated as bronchial asthma. Upper airway obstruction is often associated with obstruction to air flow. Acute (epiglossitis, angioneurotic edema, trauma etc.) obstruction can lead to acute asphyxia and even death. Elongated uvula is relatively an uncommon condition. Upper airway obstruction is often a missed complication of such a rare condition.

## CASE REPORT

A 50-year-old male presented with history of dyspnoea of sudden onset with choking sensation, waking him up at midnight, accompanied by non-productive brassy cough. Dyspnoea was more in the supine position gradually progressing to inspiratory stridor. There was a past history of similar episodes six to eight times in the last 24 months. The patient gave a history of being absolutely asymptomatic between the two episodes not even day- time sleepiness. During every episode he received antibiotics for respiratory tract infections and was treated with oxygen, bronchodilators, nebulizers and orally administered steroids. However, he was unresponsive to all that treatment given to him. A lifelong non-smoker and non-alcoholic, he had no past medical/family history. On physical examination tachypnoea and stridorous respirations were noted. There was a history of stridors that would resolve spontaneously in sitting position and re-appear in supine position. Clinical examination revealed no wheezing or rhonchi all over the chest and the patient, otherwise, had normal respiratory and cardiovascular systems. There was no clubbing or cyanosis found. Clinical examination of the thyroid gland revealed no abnormality. Except for tachypnoea, the patient's vital signs were stable.

**Figure 1:**
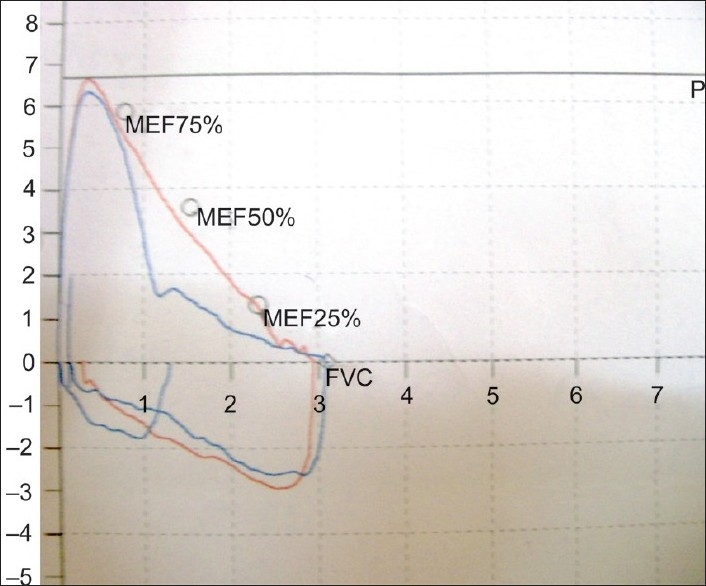
Flow-volume loop showing flattening of inspiratory flow- volume curve

A flow-volume loop study showed flattening of the inspiratory loop indicative of ‘Variable Extrathoracic Upper Airway Obstruction’ [Figure 1, Table 1]. Empey's index was 11.69 and FEF_50_%/FIF_50_% ratio (ratio of flow at 50% of expiration and 50% of inspiration) was 1.27. Upper airway obstruction (UAO) can be diagnosed either by inspecting the pattern of flow volume loops or by calculating following indices. FEV_1_/PEF ratio is known as Empey's index. UAO is indicated by FEV_1_/PEF≥8.

**Table 1:** Spirometry values

	Predicted value	Test value	Percentage (%)
FEV_1_	2.52 lit.	2.22 lit.	88
FVC	3.09 lit.	2.78 lit.	90

FEF_50_/FIF_50_

FEF_50_/FIF_50_ = 1 fixed UAO

FEF_50_/FIF_50_ > 1 variable extrathoracic UAO

FEF_50_/FIF_50_ < 0.3 variable intrathoracic UAO

As flattened inspiratory loop confirmed the upper airway compromise, the patient was subjected to flexible laryngoscopy. Laryngoscopy revealed an unusually long uvula touching the lingual surface of epiglottis especially in the supine position [Figure 2a and b].

**Figure 2a:**
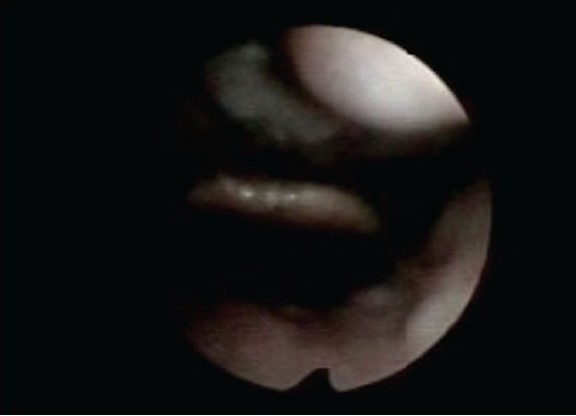
Uvula on sitting position

**Figure 2b:**
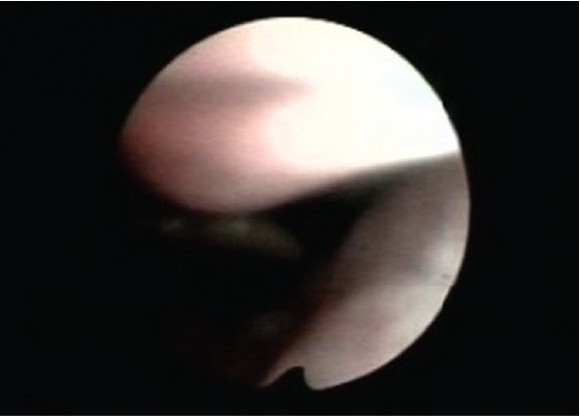
Uvula touching epiglottis in supine position

Assessment of the vocal cords under local anesthesia was normal. Local examination with flexible scope also revealed no obvious obstructive site or laxity of pharyngeal or hypopharyngeal muscles or other part of soft palate except elongated and lax uvula. Subsequently Laser uvulectomy was performed. The excised specimens were sent for histological analysis. Histopathological examination revealed no evidence of any pathology or malignancy. The patient was discharged on the second post-operative day. Three months later the patient remained asymptomatic and he is under no medications. Follow-up spirometry was suggestive of normal flow volume loops [Figure 3a and 3b].

**Figure 3a:**
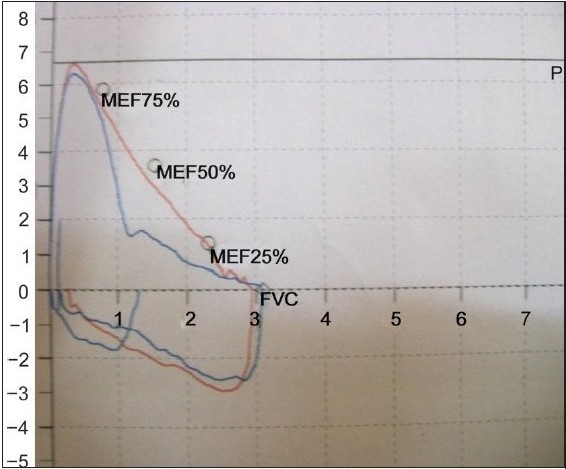
Pre-op flow-volume loop

**Figure 3b:**
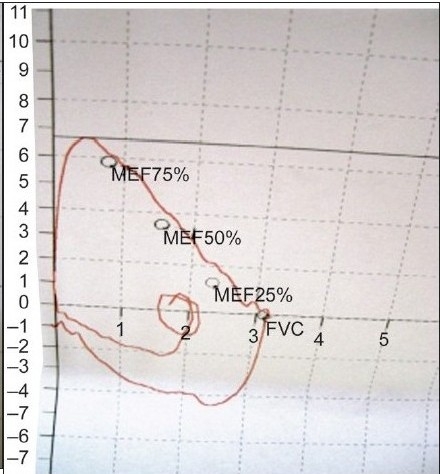
Post-uvulectomy F-V loop

## DISCUSSION

An elongated uvula is relatively a rare condition. A majority of the patients with upper airway obstruction present with obstructive symptoms that invariably mimic asthma. Nevertheless, there have been reports of cases being treated unsuccessfully as bronchial asthma.[1] Patients with symptoms suggestive of upper airway obstruction may be asymptomatic but the manifestations may range from subtle hoarseness to life threatening stridors. Particularly dyspnoea should be considered a key clinical indicator and omnious sign of upper airway compromise. Though elongated, uvula is less likely to cause life threatening airway compromise but it can definitely lead to poor quality of life due to chronic brassy cough and sleep disturbances. Management of the condition, advocated in literature, is almost exclusively surgical excision and is curative. When symptoms suggest upper airway obstruction, differential diagnosis should include tracheal masses or thyroid swelling/thyroid cancer.[2] Differential diagnosis of intra-tracheal masses should include papilloma, enchondroma, osteoma, amyloid deposits and malignancies such as thyroid carcinoma invading the trachea, chondrosarcoma, squamous cell carcinoma, adenoid cystic carcinoma, or lymphoma.[3] Another differential diagnosis for upper airway obstruction is retropharyngeal hematoma after traumatic injury.[4]

Retropharyngeal hematoma is a rare condition but may cause life-threatening airway compromise. As far as upper airway obstruction is concerned a number of reports have described patients with laryngeal dysfunction and paradoxical vocal cord motion on inspiration associated with stridor. These patients may demonstrate acute distress and require emergency endotracheal intubation or tracheostomy. Patterson *et al*. named such cases in which no organic abnormality could be found as “Miinchausen's stridor.” Vocal cord dysfunction may also present as wheezing which mimicks asthma.[5] Early detection of upper airway obstruction may help physicians avoid potentially unnecessary therapy with systemic steroids and endotracheal intubation. Upper Airway Obstruction can very easily be diagnosed by flow-volume studies. During Forced expiratory manoeuvre from TLC, the maximum flow achieved during the first 25% of the FVC depends on patient's effort. Flow rates during the remaining 75% of FVC maneuver are effort-independent and determined by the mechanical properties of the lungs.[7] In case of fixed upper airway obstruction, the F-V loop shows plateau (flattening) during both inspiration and expiration. In case of variable extra-thoracic obstruction there is an increase in turbulence of inspiratory flow and plateau of inspiratory curve. Marked reduction in expiratory flow, with relative preservation of inspiratory flow, is seen in variable intra-thoracic airway obstruction.

As UAO worsens, peak expiratory or inspiratory flows are affected early, while the FEV_ 1_ is affected only in the late stage. Thus, spirometry alone is often not helpful in detecting the presence of UAO.[6] This happened with our patients with a normal FEV_1_ per cent predicted in all. Distortion of the flow volume curve is more helpful in suggesting UAO. Notably in the cases with UAO, the FEF_50_%/FIF_50_% is found to be high, suggesting a more predominant effect on inspiration. This is expected with variable extrathoracic UAO.[7] Early detection of Upper Airway Obstruction by lung function tests can make the clinician alert to the potential for the life threatening upper airway compromise and can help avoiding unnecessary medications and endo-tracheal intubations. Moreover, recognition of upper airway obstruction may have important therapeutic implications as in this particular case laser uvulectomy offered a total cure.

Studies have proven there is no statistically significant difference in the benefit derived from laser palatoplasty when compared with uvulectomy with punctate palatal diathermy. There is a statistically significant difference in the degree of pain after the two procedures - the laser palatoplasty group has considerably more pain in the post-operative period. Uvulectomy, with punctuate palatal diathermy, is safer and has the potential to reduce morbidity and cost when compared with laser palatoplasty.[8]

## CONCLUSION

Upper airway obstruction can easily be detected on spirometry by abnormal (distorted) flow-volume loops. Early recognition of upper airway obstruction helps clinicians avoid tracheostomy. The recognition of upper airway obstruction may have important therapeutic implications as, after accurate diagnosis, patient can be offered a much less invasive procedure/surgery leading to a total cure.
